# Transcriptome analysis of *Brucella abortus* S19∆*per* immunized mouse spleen revealed activation of MHC-I and MHC-II pathways

**DOI:** 10.1099/acmi.0.000082

**Published:** 2019-12-02

**Authors:** Khushal Singh Solanki, Ravi Kumar Gandham, Prasad Thomas, Pallab Chaudhuri

**Affiliations:** ^1^​ Division of Veterinary Biotechnology, ICAR-Indian Veterinary Research Institute, Izatnagar, Bareilly, Uttar Pradesh, 243122, India; ^2^​ National Institute of Animal Biotechnology, Opp. Journalist Colony, Near Gowlidoddy Extended, Q City Road, Gachibowli, Hyderabad, Telangana 500032, India; ^3^​ Division of Bacteriology and Mycology, ICAR-Indian Veterinary Research Institute, Izatnagar, Bareilly, Uttar Pradesh, 243122, India

**Keywords:** *Brucella abortus*, vaccine, perosamine synthetase gene mutant, RNA-sequencing (RNA-seq)

## Abstract

The mouse (*Mus musculus*) has been extensively used for studying brucellosis, regarding pathogenesis, immunity and the evaluation of vaccines and therapeutics. In this work, RNA-seq was applied to explore the immunological potential of a live *Brucella abortus* S19∆*per*, a perosamine synthetase gene mutant of *B. abortus* S19. Comparison of transcriptome data was carried out for identifying differentially expressed genes among PBS (control) and *B. abortus* S19∆*per* immunized mice at 15 days post-immunization. Functional analysis revealed 545 significant differentially expressed genes related to mouse immunity. Specific activation of MHC-I and MHC-II antigen-processing pathways were identified as the highly enriched pathways based on Kyoto Encyclopedia of Genes and Genomes annotation. Other major immune response pathways regulated within the host were NF-kappa B signalling, chemokine signalling, T-cell receptor pathway, apoptosis, TNF signalling and nucleotide-binding oligomerization domain-like receptor signalling. These data provided new insights into the molecular mechanisms of *B. abortus* S19∆*per*-induced immune response in mice spleen that might facilitate the development of a highly immunogenic vaccine against brucellosis.

## Data summary

Mouse (*Mus musculus*) genome (FASTA format) and transcript annotations (GTFformat – Mus_musculus.GrCm38.83.chr.gtf.gz) was downloaded from the Ensembl genome browser (GenBank assembly accession: GCA_000001635.8).

## Introduction

Brucellosis is a global zoonotic disease causing chronic debilitating illness in humans and huge economic losses of 3.4 billion USD per year to dairy farmers in India [1]. India is now the world’s leading milk producer and hosts about 20 % of the world livestock population [2]. There is a wide prevalence of brucellosis ranging from 6.5 to 16.4 % in the country’s different livestock species [3]. In India, the brucellosis prevalence rate in cattle and buffaloes has been reported as 8.3 and 3.6%, respectively [4]. Earlier reports about brucellosis in small ruminants showed a 7.9 % prevalence rate in sheep and 2.2 % in goats [5]. The threat of brucellosis is severe in rural areas where India’s more than 65 % population lives in close contact with the large domestic animal population (512.05 million approx.). The poor living conditions, low socio-economic condition and lack of quality health services also aggravate the problem. Brucellosis is an occupational hazard for Indians who are associated with animal-husbandry activities. The higher sero-prevalence of this disease has been found in India among laboratory workers (20.0 %), dairy farmers (10.5 %), animal attendants (8.8 %), veterinary pharmacists (7.9 %), abattoir workers (6.4 %) and field veterinarians (6.3 %) [6].

Vaccination is an effective and economic way for controlling bovine brucellosis, as slaughtering of infected animals is not an option in the Indian scenario. The slaughter of cows and its progeny is banned in most of the Indian states [7]. Therefore, dairy farmers need to keep the infected dairy at farms that may act as a potential source of infection to healthy cattle as well as livestock owners. So, mass vaccination is possibly the only effective and economic way to control and eradicate bovine brucellosis in India. The World Health Organization (WHO) recommended *Brucella abortus* strain 19 vaccine to be used for animal vaccination in India and many other countries. But, S19 strain possesses several drawbacks as it may cause abortion in adult animals, possesses residual virulence and interferes with clinical diagnosis of the disease [8–11]. Our previous study reported perosamine synthetase deletion (*per*) mutant of *B. abortus* S19 named *B. abortus* S19Δ*per,* which exhibits intermediate rough phenotype with truncated lipopolysaccharide (LPS) with immunogenic properties similar to the parent strain S19. This property of attenuation has made S19**Δ** *per* a safer vaccine candidate as an alternative to the S19 vaccine [12].

To further proceed, a comprehensive understanding of immune response is necessary to design similar novel and effective vaccines. Experiments on brucellosis are generally avoided in natural hosts due to ethical, economical and practical concerns. Laboratory animals such as a mouse (*Mus musculus*) has become the standard model of brucellosis research owing to the availability of inbred strain, knockout mice, transgenic mice and better understanding of their biology and immunology [13]. RNA-sequencing (RNA-seq) is a recently developed high-throughput sequencing approach, which has several advantages over microarray in gene-expression profiling. For RNA-seq-based analysis, prior information about target sequences are not mandatory. Also, the expression-level identification is more accurate, as it is directly based on the digital count of the transcript. The microarray-derived data only provided a limited view of *Brucella* gene expression inside macrophages [14–16]. In the present study an RNA-seq-based approach was applied to gain deeper understanding of the protective mechanism in host immunized with *B. abortus* S19Δ*per* by investigating and comparing the transcriptome of spleen from mock-immunized (PBS inoculated/control) and *B. abortus* S19Δ*per*-immunized Swiss albino mice.

## Methods

### Animal and ethics

Twelve healthy female Swiss albino mice, 4–6 weeks age, weighing not less than 18 g were obtained from the Laboratory Animal Research Facility, ICAR-Indian Veterinary Research Institute (ICAR-IVRI) and divided into two groups of six mice each. All experiments in mice were done with permission and in accordance to the Institute Animal Ethics Committee (IAEC), ICAR-IVRI.

### Immunization of mice and tissue collection

*Brucella abortus* S19 ∆*per* were grown on Tryptose Soya Agar (TSA) at 37 °C for 48 h. The organism was suspended in PBS solution followed by incubation in TSA for enumeration of c.f.u. After 2 weeks of acclimatization, each mouse of group 1 (immunized) was intraperitoneally (i.p.) inoculated with 5×10^5^ c.f.u. of ∆*per* in 200 µl PBS. The mice in group 2 (control) were injected i.p. with 200 µl PBS alone and served as control or mock immunized.

At day 15 post-immunization (p.i.), all mice were euthanized humanely. Pieces of spleen harvested from three mice of the same group were pooled, washed with sterile 1× PBS, sliced into cubes of 2–3 mm. These pooled spleen samples hence represent two biological replicates each from both immunized and control mice, respectively. Spleen samples were immediately submerged in RNAlater RNA Stabilization reagent (Qiagen) @ 1 ml per 100 mg tissue in appropriate size RNase free collection vessel for RNA sequencing. Spleen samples preserved in 10 % neutral buffered formalin were processed for immunohistochemical (IHC) and immunofluorescence test (IFT).

### Brucella detection in splenic tissues

Formalin-fixed and paraffin-embedded tissue sections of spleen were mounted on poly-l-Lysine pre-coated slides (Sigma, USA) followed by deparaffinization in xylene, rehydration in graded ethanol, and quenching of endoperoxidase activity using 3 % H_2_O_2_ in methanol. Antigen retrieval was done in heat-mediated antigen retrieval solution according to the manufacturer’s instructions (Vector labs, USA). Then, 2.5 % normal non-immune goat serum (Vector labs) was used to block non-specific sites for 1 h at room temperature. For, both IHC and IFT, sections were incubated with rabbit anti-*brucella* hyper-immune serum (1 : 20) (supplied from the Division of Biological products, ICAR-IVRI) as primary antibodies. For IHC, sections were incubated with goat anti-rabbit IgG (whole molecule)-peroxidase conjugate (1 : 200) (Sigma, USA) and developed using a DAB (3,3′-diaminobenzidine tetrahydrochloride) enhanced liquid-substrate system (Sigma, USA). Immunostained sections were counter stained with Mayer’s hematoxylin (Sigma, USA) and mounted with tissue mounting medium (CC/ Mount, Sigma, USA). For IFT, goat anti-rabbit IgG (whole molecule) – FITC conjugated secondary antibodies (Sigma, USA) was used (1 : 40) followed by mounting using Fluoroshield with DAPI (4′,6-diamidino-2-phenylindole) (Sigma, USA) and viewed under fluorescent microscope (Nikon Eclipse Ti-S, Japan).

### RNA extraction, library preparation and sequencing

Total RNA was extracted from splenic tissues (representing two each biological replicate from immunized and control mice, respectively) using TRIzol reagent (QIAzol^R^Lysis reagent, Qiagen, USA) and then purified using RNeasy mini kit (Qiagen, USA). RNA was quantified using Nanodrop 1000 spectrophotometer (Thermo Scientific, USA) and Agilent 2100 Bioanalyser using Agilent RNA 6000 Nano kit, respectively. The cDNA library was prepared using low sample (LS) protocol of ‘Truseq RNA library prep kit v2 (#15026495 Rev. F, ILLUMINA proprietary, USA) followed by quality check using an Agilent 2100 Bioanalyzer. The cDNA library of all samples (four) were sequenced to generate 150 bp paired-end reads on IlluminaNextSeq 500 sequencer (Illumina, USA), according to the manufacturer’s recommendations. All steps of RNA sequencing were performed at Sandor life Sciences Pvt., Hyderabad. The RNA-seq data have been submitted to the NCBI Gene Expression Omnibus (GEO) online database (http://www.ncbi.nlm.nih.gov/geo/) [17, 18] with experiment series accession number GSE121040.

### Read mapping, identification of differentially expressed (DE) genes and functional annotation

In-house perl scripts were used for the removal of adapters and trimming of low-quality bases from the end of the raw reads. The initial processed reads were further trimmed using PRINSEQ [prinseq-lite.pl [19] to have a mean quality >=Q25 (mean phred score >25) and a minimum length of 50 nucleotides. The quality of raw and processed reads was also checked using FASTQC tool (https://www.bioinformatics.babraham.ac.uk/projects/fastqc/). The mouse (*Mus musculus*) genome (FASTA format) and transcript annotations in gtf format (Mus_musculus.GrCm38.83.chr.gtf.gz) was downloaded from the Ensembl genome browser. The gtf file was modified to extract only the exon annotations. RSEM (RNA-Seq by Expectation Maximization) [20] was used to extract the reference transcripts from the genome in combination with modified gtf file by running the ‘rsem-prepare-reference’ command. The processed reads (R1 and R2) were aligned seperately with the reference transcripts using Bowtie 2 alignment program [21]. Further, rsem-calculate-expression script was run for each of the alignments to calculate expression level from reads (transcript abundances or RNA-Seq quantification) in terms of ‘expected counts’. The expected counts estimated by RSEM were fed into different DE package tools, such as DESeq2 [22], edgeR [23] and EBSeq [24] to identify differentially expressed genes between different groups.

Functional annotation of genes was performed using online bioinformatics tools g:Profiler [25] (http://biit.cs.ut.ee/gprofiler/). The tool performs statistical-enrichment analysis to find over-representation of information like gene ontology (GO) terms such as biological processes, molecular functions, cellular components and Kyoto Encyclopedia of Genes and Genomes (KEGG) pathways.

## Results

### *B. abortus* S19 ∆*per* detection in spleen tissues

**Fig. 1.**
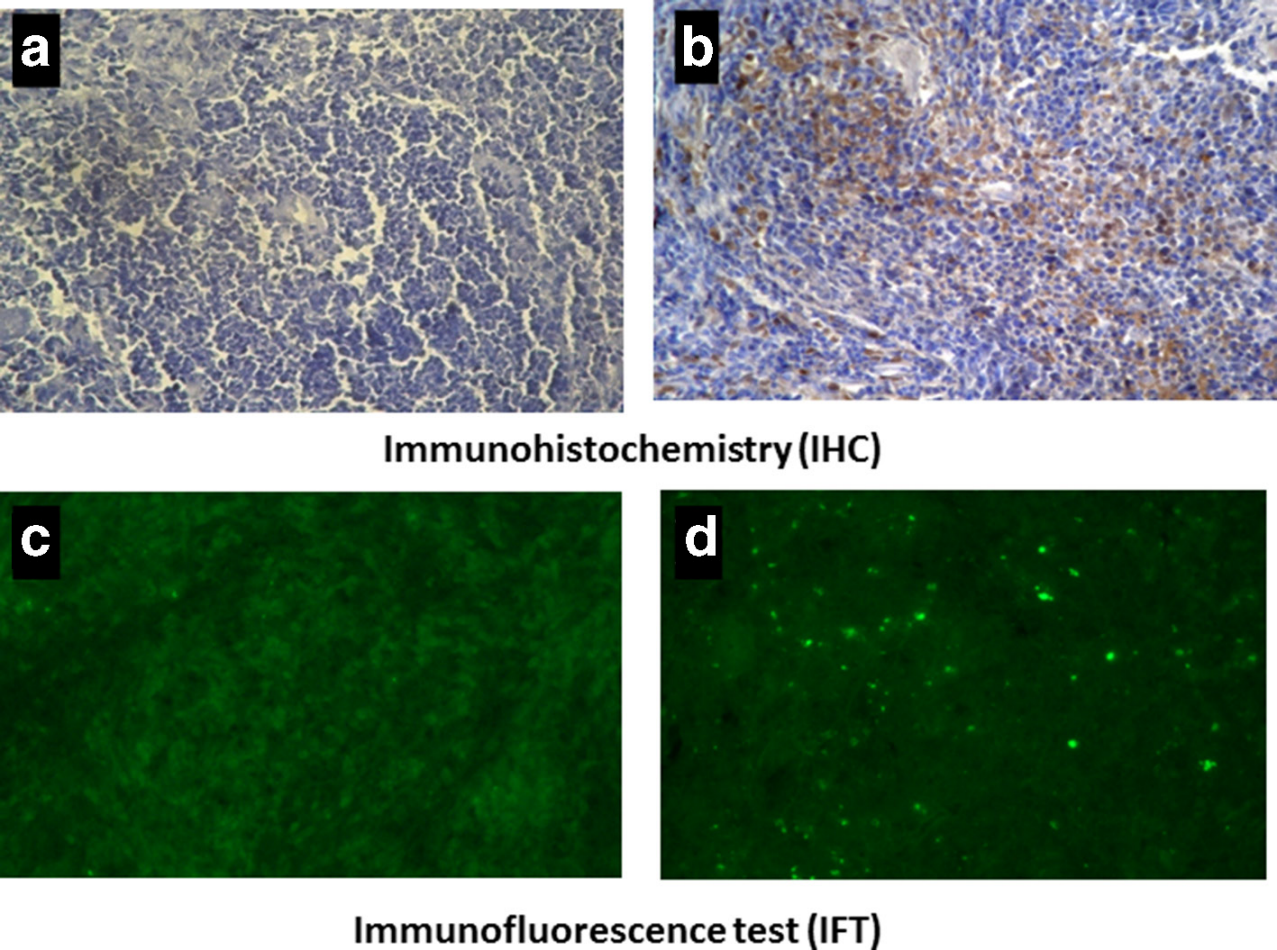
IHC and IFT staining of a mouse spleen immunized with *B *. *abortus* S19 ∆*per.* IHC (Fig. 1 above - a and b) and FITC-labelled immunofluorescent (Fig. 1 below - c and d) staining of a spleen of Swiss albino mice at day 15 p.i. with 5×10^5^ c.f.u. of *B. abortus* S19 ∆*per and* PB controls. Both, IHC (b) and IFT (d) tests showed positive results in the form of the golden-brown deposition of DAB (3,3′-diaminobenzidine tetrahydrochloride) within macrophages and lymphocytes and apple green fluorescence, respectively. The PBS control (a and c) did not show any positive signal.

At day 15 p.i. of *B. abortus* S19 ∆*per*, both IHC and IFT tests showed positive results in the form of the golden-brown deposition of DAB (3,3′-diaminobenzidine tetrahydrochloride) within macrophages and lymphocytes and apple green fluorescence, respectively, in spleen (Fig. 1).

### Differential expression between Δ*per* and mock-immunized mouse spleen

The extracted RNA from mice spleen (biological replicates) was of good quality displaying a 260/280 ratio >1.8 and having optimum RNA integrity – RIN >7, which is a basic requirement for expression studies [26]. The quality of cDNA libraries assessed confirmed library insert sizes to be 270±10 bp. A total of four cDNA libraries were prepared, of which two each (biological replicates) represented mutant (* B. abortus* S19Δ*per*) inoculated mouse spleen samples (immunized) and PBS (control) inoculated mouse spleens, respectively. cDNA libraries generated an average number of 35.07 and 45.03 million raw paired end (PE) reads for Δ*per* and mock-control samples, respectively. Filtration and trimming of raw-sequence reads yielded a mean of 37.58 million processed reads (range: 30.51 million to 51.79 million reads) per individual RNA-seq library. Read statistics of obtained and processed reads are shown in Table S1, available in the online version of this article.

The obtained reads were finally mapped to the *Mus musculus* genome using RSEM (RNA-Seq by Expectation Maximization) for evaluating gene expression. A total of 1917 differentially expressed genes (DEGs) were identified using different DE packages, such as DESeq2, edgeR and EBSeq, out of which 968 and 949 genes were up- and down-regulated, respectively, in the *B. abortus* S19Δ*per* immunized mice spleen relative to the un-immunized control sample.

### Function annotation of differentially expressed genes

**Fig. 2.**
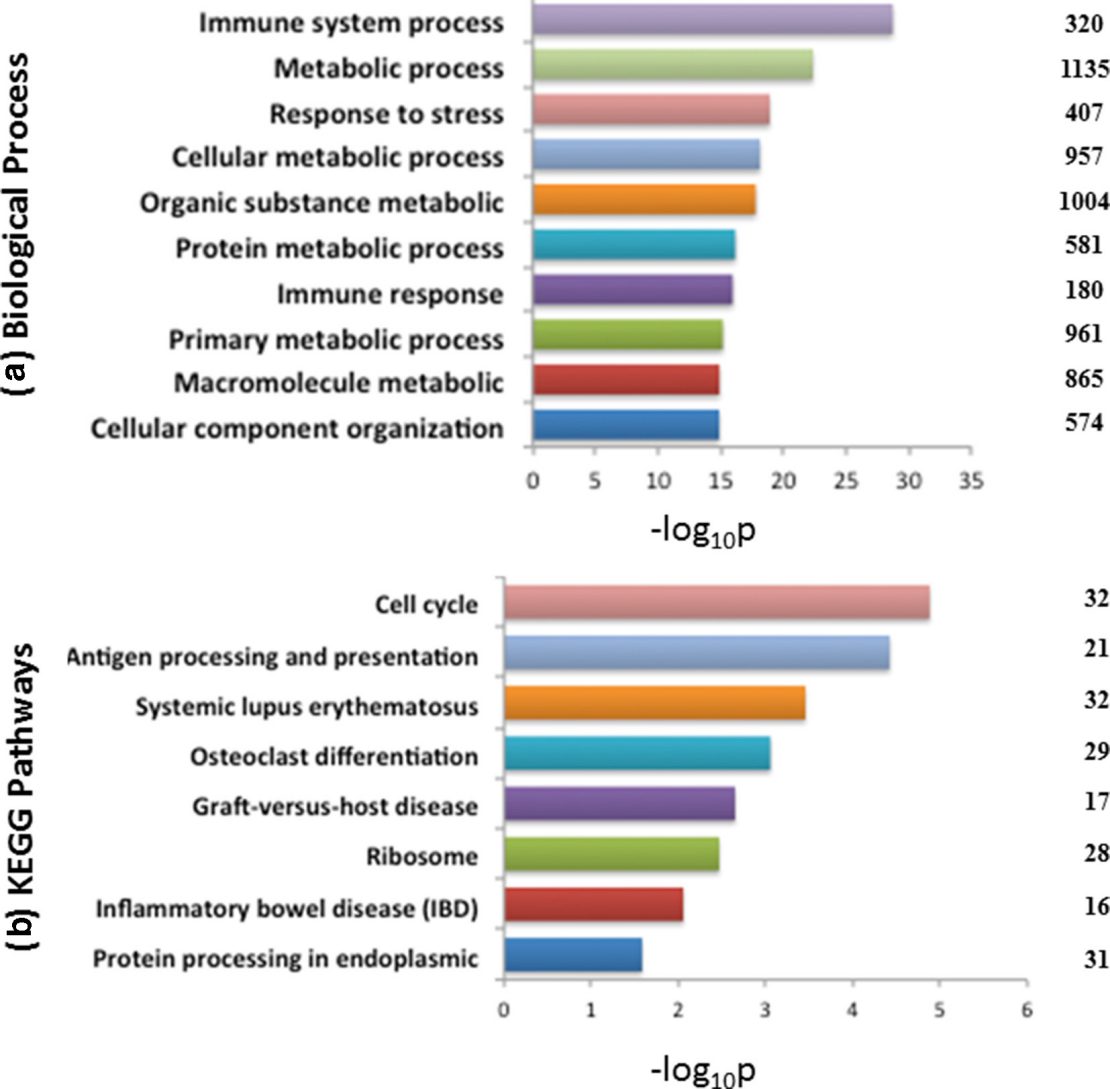
Enriched ontological and KEGG pathway analysis of DEGs. The most significantly enriched (*P*<0.05) GO terms (biological processes) (a) and KEGG pathways (b) of Δ*per*-immunized mice vs. control (uninfected) at day 15 p.i. by g:Profiler are shown. The numbers shown at the right side of the bar represent the no. of DEGs involved in the GO term. The enrichment *P*-value of each term was transformed to a –log(*P*-value).

GO enrichment analysis of 1917 DEGs using g:Profiler, yielded a total of 613 significant GO terms. Among them, 291, 33 and 50 were related to biological processes, molecular functions and cellular components, respectively. The significant enrichment of DE genes in the cluster of biological processes was seen for immune system process (GO: 0002376), metabolic process (GO: 0008152), response to stress (GO: 0006950), cellular metabolic process (GO: 0044237) organic substance metabolic process (GO: 0071704) and protein metabolic process (GO: 0019538). A total of 320 and 180 transcripts whose encoded proteins are known to be involved in immune system processes (*P*-value=2.02E-29, GO: 0002376) and immune response (*P*-value=1.17E-16, GO: 0006955), respectively, exhibited significant enrichment in Δ*per* in comparison to the control group (Fig. 2a). KEGG annotation revealed DEGs to be enriched in pathways pertaining to cell cycle (*P*-value=0.0000132, KEGG: 04110), antigen processing and presentation (*P*-value=0.0000379, KEGG: 04612) and protein processing in endoplasmic reticulum (*P*-value=0.0262, KEGG: 04141) (Fig. 2b). The list of significant GO terms (biological process and molecular functions) and top KEGG pathways, identified by g:Profiler are represented in Table S2.

**Fig. 3.**
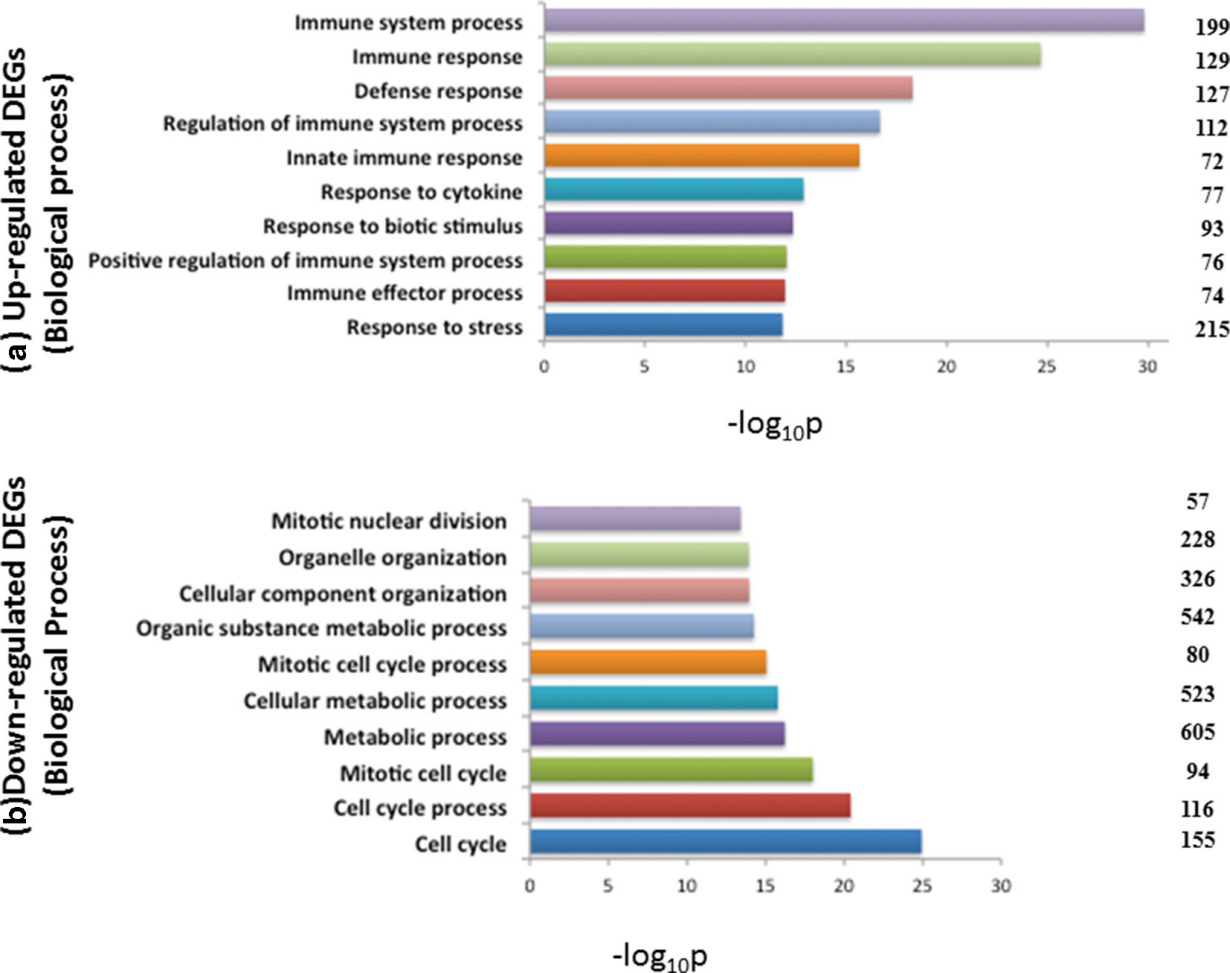
Biological processes involved by up-regulated (a) and down-regulated (b) DEGs. Top enriched biological processes targeted by up-regulated (a) and down-regulated (b) DEGs, identified in comparison of RNA-seq data of *B. abortus* S19∆*per* with uninfected (control) mice. Enriched classification was determined using g:Profler. The numbers shown at the right side of the bar represent the no. of DEGs involved in the GO term. The enrichment *P*-value of each term was transformed to a –log(*P*-value).

GO enrichment analysis of 968 up-regulated and 949 down-regulated genes using g:Profiler revealed that most of the biological processes targeted by over-expressed DEGs were related to immune response, whereas, most of the top biological processes governed by under-expressed DEGs were related to cell cycle and its associated processes and metabolism, as depicted in Fig. 3. Among the 1917 differentially expressed genes, a total of 545 significantly enriched genes were found associated to the immune-system processes (Table S3). Functional analysis using g:Profiler revealed that these immunity related genes were distributed in 998 and 42 significant GO terms belonging to biological process and KEGG pathways, respectively (Table S4).

The list of significantly enriched pathways form DEGs related to immune response identified in the KEGG database are shown in Table 1. In this study, Δ*per* immunization increased dramatically the expression of 21 genes; 20 up and only one down-regulated in antigen processing and presentation pathway (KEGG: 04612), the top most significant pathway. Among the 20 up-regulated genes, 14 genes were involved in the MHC-I pathway and 06 genes were involved in MHC-II pathway. Under chemokine signalling pathway, most of proinflammatory chemokines or their receptors were up-regulated (Table 2).

**Table 1.** List of significantly enriched pathways from DEGs related to immune response. *In silico* prediction of DEGs, identified in the comparison of RNA-seq data of *B. abortus* S19∆*per* immunized mice with control (uninfected) at day 15 p.i. into significantly enriched immunity related KEGG pathways

S. No.	Pathway	*P*-value	No. of DEGs	No. of up-regulated DEGs	No. of down-regulated DEGs	Up-regulated DEGs	Down-regulated DEGs
1	Antigen processing and presentation (KEGG:04612)	1.18E-09	21	20	01	*H2-M3,PSME1,TAPBP,TAP2,TNF,CD74,KLRD1,* *H2-AA, TAP1,H2-DMB2,H2 DMA,CD8B1,CD8A,* *IFNG,H2-Q7,H2-K1,H2-BL,H2-AB1,PSME2,H2-DMB1*	*H2-T24*
2	NF-kappa B signalling pathway (KEGG:04064)	1.47E-06	19	13	06	*PLCG1,VCAM1,CFLAR,CD14,BCL2A1B,BCL2A1A,* *TNFAIP3,LAT,TNF,ZAP70, CCL4,IL1B,CXCL2*	*NFKB1,NFKB2,PIDD1,PARP1,* *IL1R1,GM21541*
3	Chemokine signalling pathway (KEGG:04062)	4.91E-05	25	13	12	*STAT2,CXCL16,CCR5,STAT1,CXCR6,* *CXCR3,CCL4,CXCL2,CCL24,CCL5,CXCL11,* *CXCL9,CCL8*	*NFKB1,MAP2K1,MAPK1,GSK3A,* *HRAS,NCF1,CXCR5,CCR9,CRKL,* *CXCL13,CCR6,GM21541*
4	TNF signalling pathway (KEGG:04668)	0.00022	17	13	04	*IFI47,CXCL2,SOCS3,JUNB,CCL5,VCAM1,IL1B,DAB2IP,* *IL18R1,CFLAR,FAS,TNF,TNFAIP3*	*MAP2K1, NFKB1,CASP3,MAPK1*
5	T cell receptor signalling pathway (KEGG:04660)	0.000978	16	12	04	*PLCG1,FYN,CD247,LAT,CD3D,TNF,ZAP70,CD8B1,* *CD8A,PDCD1,IL10,IFNG*	*NFKB1,MAP2K1,MAPK1,HRAS*
6	Apoptosis (KEGG:04210)	0.00169	18	09	09	*FAS,ENDOG,CFLAR,BCL2A1B,BCL2A1A,* *DAB2IP,TNF,GADD45G,CTSW*	*NFKB1,MAP2K1,MAPK1,HRAS,* *PIDD1,PARP1,CASP2,SPTA1,CASP3*
7	Nucleotide-binding oligomerization domain (NOD)-like receptor signalling pathway (KEGG:04621)	0.00194	11	08	03	*CXCL2,NOD1,CCL5,PYCARD,IL1B,CASP1,TNF,TNFAIP3*	*MAPK1,NFKB1,ERBB2IP*

p.i. stands for post inoculation

**Table 2.** *In silico* prediction of DEGs into significantly enriched pathways. *In silico* prediction of DEGs into significantly enriched antigen processing and presentation pathway (a); chemokine signalling pathway (b) and T-cell receptor signalling pathway (c).

S. No (a)	Official gene symbol	Gene description (Antigen processing and presentation pathway)	Log_2_FC
1	*IFNG*	Interferon gamma [Source: MGISymbol; Acc: MGI: 107656]	2.963021013
2	*H2-Q7*	Histocompatibility 2, Q region locus 7 [Source: MGI Symbol; Acc: MGI: 95 936]	1.672332678
3	*TAP1*	Transporter 1, ATP-binding cassette, sub-family B (MDR/TAP) [Source: MGI Symbol; Acc: MGI: 98 483]	1.464058075
4	*CD8A*	CD8 antigen, alpha chain [Source: MGI Symbol; Acc: MGI: 88 346]	1.140741908
5	*CD8B1*	CD8 antigen, beta chain 1 [Source: MGI Symbol; Acc: MGI: 88 347]	1.10820778
6	*KLRD1*	Killer cell lectin-like receptor, subfamily D, member 1 [Source: MGI Symbol; Acc: MGI: 1196275]	1.095336268
7	*TNF*	Tumor necrosis factor [Source: MGI Symbol; Acc: MGI: 104798]	0.990003736
8	*TAP2*	Transporter 2, ATP-binding cassette, sub-family B (MDR/TAP) [Source: MGI Symbol; Acc: MGI: 98 484]	0.973927258
9	*H2-DMA*	Histocompatibility 2, class-II, locus DMa [Source: MGI Symbol; Acc: MGI: 95 921]	0.957013483
10	*H2-BL*	Histocompatibility 2, blastocyst [Source: MGI Symbol; Acc: MGI: 892004]	0.898031322
11	*PSME1*	Proteasome (prosome, macropain) activator subunit 1 (PA28 alpha) [Source: MGI Symbol; Acc: MGI: 1096367]	0.808565827
12	*H2-K1*	Histocompatibility 2, K1, K region [Source: MGI Symbol; Acc: MGI: 95 904]	0.804961707
13	*H2-AB1*	Histocompatibility 2, class-II antigen A, beta 1 [Source: MGI Symbol; Acc: MGI: 103070]	0.770383259
14	*H2-M3*	Histocompatibility 2, M region locus 3 [Source: MGI Symbol; Acc: MGI: 95 915]	0.762926924
15	*H2-AA*	Histocompatibility 2, class-II antigen A, alpha [Source: MGI Symbol; Acc: MGI: 95 895]	0.720227941
16	*CD74*	CD74 antigen (invariant polypeptide of major histocompatibility complex, class-II antigen-associated) [Source: MGI Symbol; Acc: MGI: 96 534]	0.687810587
17	*TAPBP*	TAP binding protein [Source: MGI Symbol; Acc: MGI: 1201689]	0.66876859
18	*PSME2*	Proteasome (prosome, macropain) activator subunit 2 (PA28 beta) [Source: MGI Symbol; Acc: MGI: 1096365]	0.644390732
19	*H2-DMB1*	Histocompatibility 2, class-II, locus Mb1 [Source: MGI Symbol; Acc: MGI: 95 922]	0.568768704
20	*H2-DMB2*	Histocompatibility 2, class-II, locus Mb2 [Source: MGI Symbol; Acc: MGI: 95 923]	0.458075837
21	*H2-T24*	Histocompatibility 2, T region locus 24 [Source: MGI Symbol; Acc: MGI: 95 958]	−1.395089545
**S.No (b)**	**Official gene symbol**	**Gene description (Chemokine signalling pathway)**	**Log**_**2**_ **FC**
1	*CCL8*	Chemokine (C-C motif) ligand 8 [Source:MGI Symbol;Acc:MGI:101878]	3.263308066
2	*CXCL9*	Chemokine (C-X-C motif) ligand 9 [Source:MGI Symbol;Acc:MGI:1352449]	2.224042495
3	*CXCL11*	Chemokine (C-X-C motif) ligand 11 [Source: GI Symbol; Acc: MGI: 1860203]	2.122075948
4	*CCL5*	Chemokine (C-C motif) ligand 5 [Source: MGI Symbol; Acc: MGI: 98 262]	1.997847444
5	*CCL24*	Chemokine (C-C motif) ligand 24 [Source: MGI Symbol; Acc: MGI: 1928953]	1.725151475
6	*CXCL2*	Chemokine (C-X-C motif) ligand 2 [Source: GI Symbol; Acc: MGI: 1340094]	1.323049568
7	*CCL4*	Chemokine (C-C motif) ligand 4 [Source: MGI Symbol; Acc: MGI: 98 261]	1.169987071
8	*CXCR3*	Chemokine (C-X-C motif) receptor 3 [Source: MGI Symbol; Acc: MGI: 1277207]	1.082820268
9	*CXCR6*	Chemokine (C-X-C motif) receptor 6 [Source: MGI Symbol;Acc: MGI: 1934582]	0.999063481
10	*STAT1*	Signal transducer and activator of transcription 1 [Source: MGI Symbol; Acc: MGI: 103063]	0.89990532
11	*CCR5*	Chemokine (C-C motif) receptor 5 [Source: MGI Symbol; Acc: MGI: 107182]	0.846015109
12	*CXCL16*	Chemokine (C-X-C motif) ligand 16 [Source: MGI Symbol; Acc: MGI: 1932682]	0.566003653
13	*STAT2*	Signal transducer and activator of transcription 2 [Source: MGI Symbol; Acc: MGI: 103039]	0.439314708
14	*GM21541*	Predicted gene, 21541 [Source: MGI Symbol; Acc: MGI: 5434896]	−2.92287106
15	*CCR6*	Chemokine (C-C motif) receptor 6 [Source: MGI Symbol; Acc: MGI: 1333797]	−1.58175143
16	*CXCL13*	Chemokine (C-X-C motif) ligand 13 [Source: MGI Symbol; Acc: MGI: 1888499]	−1.201331282
17	*CRKL*	V-crk avian sarcoma virus CT10 oncogene homolog-like [Source: MGI Symbol; Acc: MGI: 104686]	−1.174023131
18	*CCR9*	Chemokine (C-C motif) receptor 9 [Source: MGI Symbol; Acc: MGI: 1341902]	−1.008486432
19	*CXCR5*	Chemokine (C-X-C motif) receptor 5 [Source: MGI Symbol; Acc: MGI: 103567]	−0.695670228
20	*NCF1*	Neutrophil cytosolic factor 1 [Source: MGI Symbol; Acc: MGI: 97 283]	−0.568119989
21	*HRAS*	Harvey rat sarcoma virus oncogene [Source: MGI Symbol; Acc: MGI: 96 224]	−0.544679554
22	*GSK3A*	Glycogen synthase kinase 3 alpha [Source: MGI Symbol; Acc: MGI: 2152453]	−0.532461503
23	*MAPK1*	Mitogen-activated protein kinase 1 [Source: MGI Symbol; Acc: MGI: 1346858]	−0.526774692
24	*MAP2K1*	Mitogen-activated protein kinase kinase 1 [Source: MGI Symbol; Acc: MGI: 1346866]	−0.516692525
25	*NFKB1*	Nuclear factor of kappa light polypeptide gene enhancer in B cells 1, p105 [Source: MGI Symbol; Acc: MGI: 97 312]	−0.453873719
**S. No (c)**	**Official gene symbol**	**Gene description (T cellreceptor signaling pathway)**	**Log_2_FC**
1	*IFNG*	Interferon gamma [Source: MGISymbol; Acc: MGI: 107656]	2.963021013
2	*IL10*	Interleukin 10 [Source: MGI Symbol; Acc: MGI: 96 537]	1.702910973
3	*PDCD1*	Programmed cell death 1 [Source: MGI Symbol; Acc: MGI: 104879]	1.617129947
4	*CD8A*	CD8 antigen, alpha chain [Source: MGI Symbol; Acc: MGI: 88 346]	1.140741908
5	*CD8B1*	CD8 antigen, beta chain 1 [Source: MGI Symbol; Acc: MGI: 88 347]	1.10820778
6	*ZAP70*	Zeta-chain (TCR) associated protein kinase [Source: MGI Symbol; Acc: MGI: 99 613]	1.0116642
7	*TNF*	Tumor necrosis factor [Source: MGI Symbol; Acc: MGI: 104798]	0.990003736
8	*CD3D*	CD3 antigen, delta polypeptide [Source: MGI Symbol; Acc: MGI: 88 331]	0.905417968
9	*LAT*	Linker for activation of T cells [Source: MGI Symbol; Acc: MGI: 1342293]	0.870380028
10	*CD247*	CD247 antigen [Source: MGI Symbol; Acc: MGI: 88 334]	0.654270003
11	*FYN*	Fyn proto-oncogene [Source: MGI Symbol; Acc: MGI: 95 602]	0.595868291
12	*PLCG1*	Phospholipase C, gamma 1 [Source: MGI Symbol; Acc: MGI: 97 615]	0.463807705
13	*HRAS*	Harvey rat sarcoma virus oncogene [Source: MGI Symbol; Acc: MGI: 96 224]	−0.544679554
14	*MAPK1*	Mitogen-activated protein kinase 1 [Source: MGI Symbol; Acc: MGI: 1346858]	−0.526774692
15	*MAP2K1*	Mitogen-activated protein kinase kinase 1 [Source: MGI Symbol; Acc: MGI: 1346866]	−0.516692525
16	*NFKB1*	Nuclear factor of kappa light polypeptide gene enhancer in B cells 1, p105 [Source: MGI Symbol; Acc: MGI: 97 312]	−0.453873719

Note: All up-/down-regulated (Log_2_FC) genes had *P*<0.05, Padj.<0.05 and FDR<0.05. FC stands for ‘fold change’. Fold change’ is the magnitude of up- or –down regulation of each gene. The ‘+’ values of fold change indicate up-regulation and ‘–’ values indicate down-regulation. Official gene symbols are given according to their log_2_FC in decreasing order.

## Discussion

The murine model has been found useful in microarray and RNA-seq-based transcriptome profiling for studying brucella: host interactions [27, 28]. In this work, RNA-seq was applied to investigate the immunization-related gene-expression patterns. The study was targeted to gain deep insight into the immunological potential of *B. abortus* S19∆*per in vivo* in a murine model on day 15 p.i. The spleen is the secondary lymphoid organ where innate and adaptive immune responses can be well displayed.

**Fig. 4.**
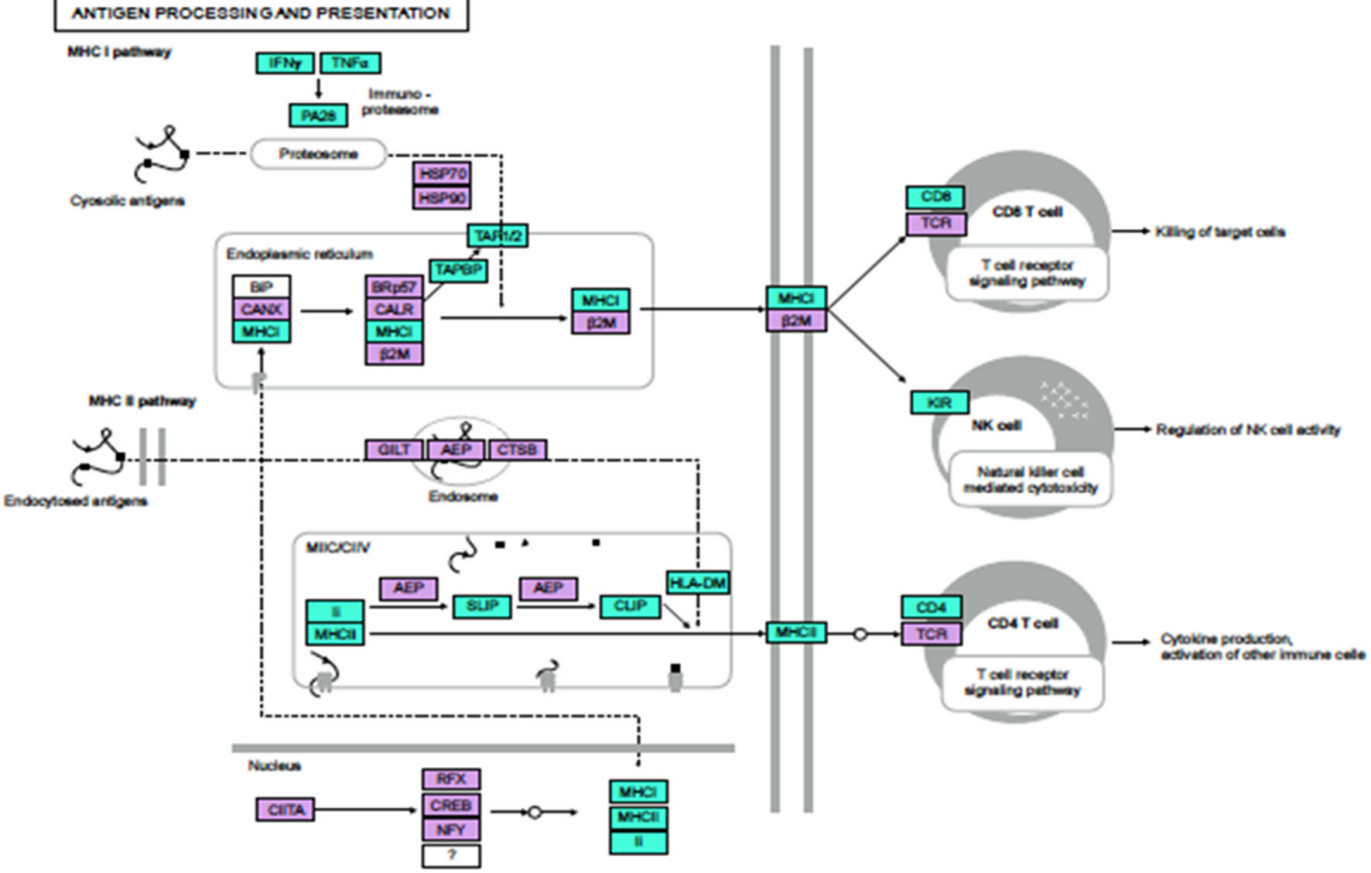
Antigen processing and presentation (KEGG) pathway regulation at day 15 post-* B. abortus* S19∆*per* immunization. Genes associated with antigen processing and presentation pathway at day 15 post-* B. abortus* S19∆*per* immunization. The pathway associated significant DEGs relative to control (uninfected) samples are highlighted in colour. Green: significantly increased expression: Purple: unchanged expression.

In ∆*per*-immunized mouse spleen, 21 differently expressed transcripts were mapped to antigen processing and presentation pathway (KEGG: 04612), the most significant pathway that has an important implication in *Brucella* immunity (Table 2a). Most of the up-regulated genes [24] were related with MHC-I processing pathway than MHC-II pathway with 06 up-regulated genes (Fig. 4). The bacterial vaccine organisms are internalized by host phagocytic cells via different entry mechanisms. In the conventional paradigm, MHC class-II binding peptides are derived from exogenous antigen, which is endocytosed and degraded in the lysosomal/endosomal compartment. Class-II MHC molecules are synthesized and transferred from endoplasmic reticulum (ER) to endosomal compartment where derived peptides are attached with their antigen-binding pocket. This class-II MHC–peptide complex is presented on the cell surface to be recognized by CD4^+^ T cells [29]. On the other hand, MHC class-I molecules present antigenic fragments to CD8^+^ T cells, which have an important role in the clearance of *Brucellae*, probably by lysing-infected macrophages [30]. In MHC-I antigen processing pathway, antigenic peptides are degraded in the cytoplasm by proteasome, then translocated into the ER and loaded onto MHC-I molecules with the help of several protein components. PA28 is a hetero-hexameric proteasome activator ring, which binds to one or both ends of the 20 s proteasome, and therefore, increases its catalytic activity leading to changes in substrate cleavage and generating more MHC class-I-presented peptides [31–33]. In this study, the *IFNG*, *PMSE1* and *PMSE2* encoding IFN-γ, proteasome activator subunit 1 (PA28 α) and protesome activator subunit 2 (PA28 β), respectively, were up-regulated, which suggested the activation of MHC-I pathway in ∆*per* immunized mice spleen at day 15 p.i. (Table 2a).

Our results are also substantiated by an earlier study that reports the role of IFN-γ in modifying proteasome activity (replacing proteasome by immunoproteasome) by up-regulating PA28 α and PA28 β in *Listeria monocytogenes* or lymphocytic choriomeningitis infected mice liver within 7 days [34]. In addition, transporter proteins (TAP1 and TAP2) and TAP-binding protein (TAPBP), both involved in translocation of proteasome-degraded peptides in ER lumen; and cell-surface expressing class-I MHC molecules – *H2-Q7*, *H2-BL*, *H2-K1*, *H2-M3* – were also up-regulated, strongly suggesting the activation of the MHC-I processing pathway following ∆*per* immunization in mice. The presented processed peptides along with MHC class-I molecules are recognized by CD8^+^ T cells [23]. Two genes, *CD8A* and *CD8B1*, encoding α and β chains of CD8 antigen, respectively, were also up-regulated (Table 2a, c).

The RNA-seq data of ∆*per* immunized mice has also shown a coordinated up-regulation of several MHC class-II antigen processing and presentation components such as H2- DMA, H2-DMB1, H2-DMB2, H2-AA, H2-AB1 and CD74, which suggested activation of MHC-II processing pathway. Upon ER translocation, the invariant chain (Ii chain or CD74) associates with the nascent α and β subunits of MHC-II (encoded by *H2-AA*, *H2-AB1*) to form MHC-II αβ dimers, preventing the binding of ER protein to MHC-II peptide binding cleft and directs MHC-II molecules to endosomal compartments [35, 36] where they are degraded by resident proteases leaving only the peptide-binding region, CLIP (class-II-associated invariant chain peptide) bound to MHC-II [37]. The mouse endosomal protein H2-DM (HLA-DM in human) then facilitates peptide exchange to release CLIP and subsequent peptide binding to the MHC-II-binding pocket [38] followed by its presentation on the surface of antigen presenting cells to be recognized by CD4^+^ T cells. In brucellosis, peptides are presented by both MHC class-I and MHC class-II molecules and therefore, induce both CD4^+^ and CD8^+^ *Brucella *-specific T cells. Similar findings with the involvement of both CD4^+^ and CD8^+^ T cells in conferring protection for *B. abortus* S19 vaccine have been reported [39].

**Fig. 5.**
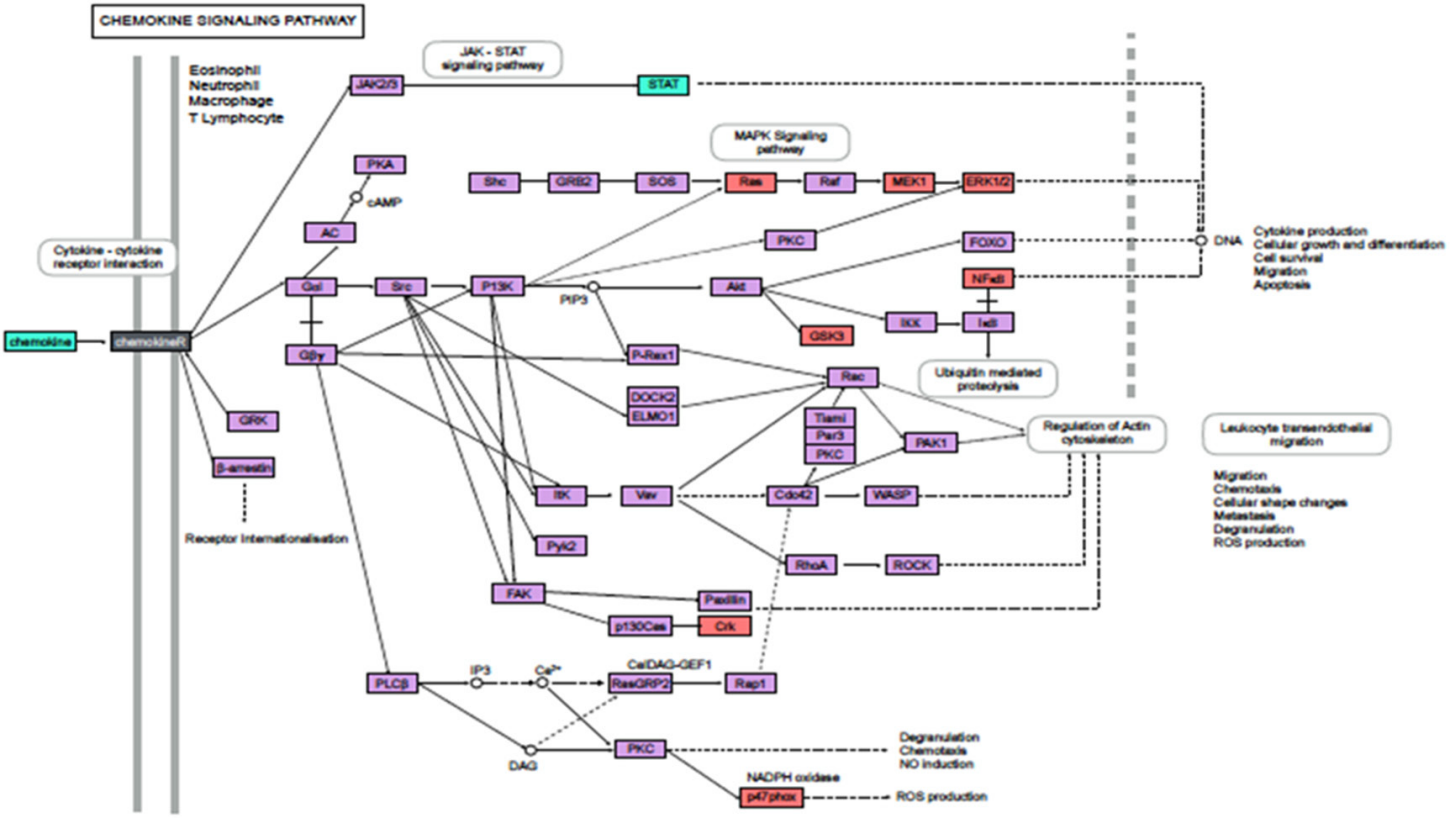
Chemokine signalling (KEGG) pathway regulation at day 15 post-* B. abortus* S19∆*per* immunization. Genes associated with chemokine signalling pathway at day 15 post-* B. abortus* S19∆*per* immunization. The pathway associated significant differentially expressed genes relative to control (uninfected) samples are highlighted in colour. Green: significantly increased expression: Purple: unchanged expression: Drack Grey: Gene detected in both up and down regulated groups.

Inflammation is a powerful protective mechanism, coordinated and controlled by cytokines and chemokines. The up-regulation in the transcription of proinflammatory chemokines transcripts such as *CCL8*, *CCL5* (RANTES), *CCL24*, *CCL4*, *CXCL9*, *CXCL11*, *CXCL2* and *CXCL16* reiterates the activation of chemokine signalling pathway and likely represents an anti-bacterial response by host cells against *B. abortus* S19∆*per* (Table 2b, Fig. 5).

In conclusion our study reports the majority of DEGs involved in *B. abortus* S19∆*per* immunized mice were up-regulated and found involved in immunity-related pathways related to antigen processing and presentation pathway, NF-kappa B signalling, chemokine signalling, T-cell receptor signalling, TNF signalling, NOD-like receptor signalling. This is indicative of the *B. abortus* S19Δ*per* role in triggering vigorous adaptive immune response by activating these genes and pathways. The RNA-seq data revealed a coordinated up-regulation of MHC-I and MHC-II processing pathways providing insights into the molecular mechanism of immune protection conferred by *B. abortus* S19∆*per* in mice at day 15 p.i.

## Supplementary Data

Supplementary material 1

Supplementary material 2

Supplementary material 3

Supplementary material 4
